# A survey on migraine attack treatment with the CEFALY^®^ device in regular users

**DOI:** 10.1007/s13760-017-0757-z

**Published:** 2017-02-09

**Authors:** Sophie Penning, Jean Schoenen

**Affiliations:** 1 Cefaly Technology, Grâce-Hollogne, Belgium; 2 Headache Research Unit, University Department of Neurology, Citadelle Hospital, Liège University, Boulevard du 12ème de Ligne, 1, 4000 Liege, Belgium

**Keywords:** Acute migraine treatment, External trigeminal neurostimulation, Cefaly^®^

A randomized, double blind, sham-controlled trial proved the efficacy and safety of external trigeminal nerve stimulation (e-TNS) with the Cefaly^®^ device (CEFALY Technology, Belgium) for the preventive treatment of episodic migraine [1]. Safety and patients’ satisfaction were confirmed by a retrospective survey of 2313 users [2].

Although many patients also report benefit from using the device during migraine attacks, only limited data is available on the efficiency of Cefaly^®^ for attack treatment. In a pilot trial of ten episodic migraine patients who treated three attacks with the device [3], total relief without rescue medication was reported in 12% of attacks, incomplete relief with rescue medication in 42.5% and no effect in 45.5%. In an open study including 16 patients, the Cefaly^®^ device was effective and well tolerated as rescue therapy for migraine attack symptoms present since at least 72 h and reduced the headache on average by 46% [4].

While awaiting the results of larger controlled trials, useful information might be obtained by interviewing migraineurs who apply the device for migraine prevention about its use during attacks and ability to reduce acute anti-migraine drug intake. We conducted, therefore, a survey on 807 Belgian, Swiss and French subjects from the Cefaly^®^ customer database who were identified as regular users because they had purchased the device and ordered new electrodes within the last year. One of us (JS) invited them by email to answer on-line an eight-item questionnaire, using the SurveyMonkey [5] service provider to implement the survey and collect the results. Confidentiality was guaranteed by fully disabling the electronic and IP addresses recordings in order to collect anonymous responses.

**Fig. 1:**
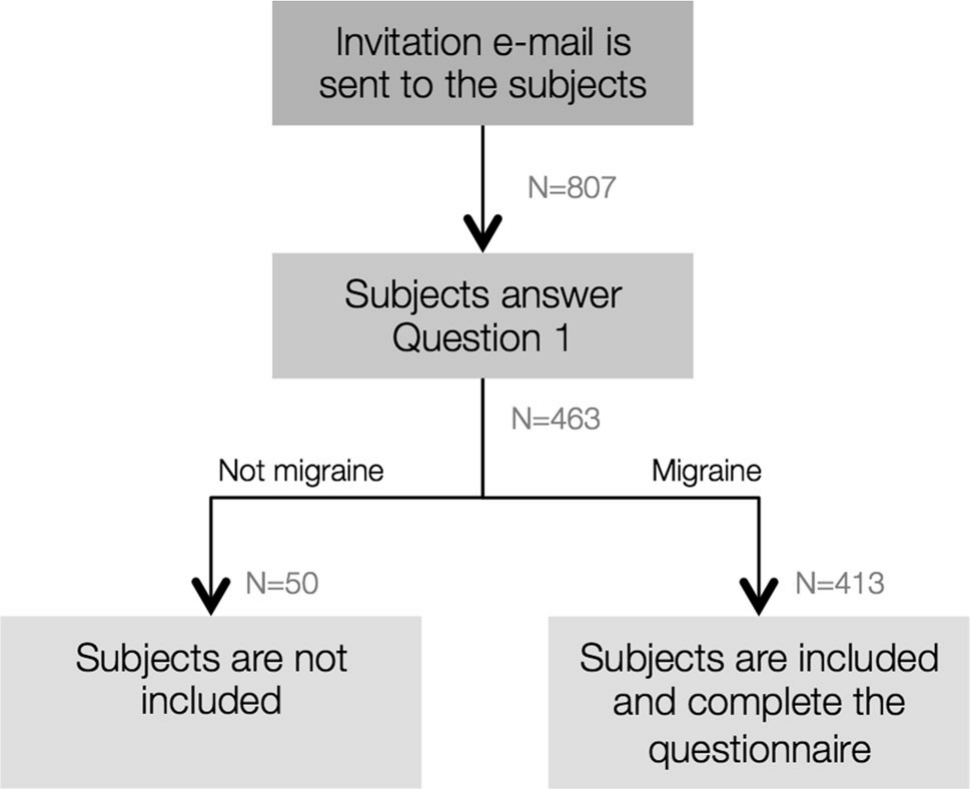
Flow chart of the study protocol

Among 463 subjects who filled in the questionnaire (57% responder rate), 413 (89.2%) who answered “yes” to the first question “You suffer from headaches. Has a physician diagnosed them as typical migraine?*”* were invited to proceed to the following questions and included in the analyses (Fig. 1). The questionnaire was designed to retrieve the following information: monthly attack frequency, use of Cefaly^®^ during an attack or reasons for non-using it, proportion of attacks treated with Cefaly^®^, proportion of Cefaly^®^-treated attacks with reduction of acute anti-migraine drug intake, class of drugs with reduced intake.

The primary outcome measure was the mean number of acute anti-migraine drug intake avoided per month per patient thanks to the use of Cefaly^®^. Secondary outcome measures were: percentage of subjects using the device during an attack, percentage of attacks treated with the device, percentage of Cefaly^®^-treated attacks with reduction of acute anti-migraine drugs.

The results are displayed in Table 1. Among the 413 regular device users for prevention, 88.6% also used it as an acute treatment in 71.8% of their attacks. In 42.6% of these attacks the use of Cefaly^®^ was accompanied by reduced intake of acute anti-migraine medications. For the total cohort, Cefaly^®^ allowed to reduce acute migraine drug intake on average in 2.93 attacks per month per subject. If only those 366 subjects using the device for attack treatment are considered, this number increases to 3.31 attacks per month per subject. This represents the lower bound of the actual numerical reduction in drug intake, since for one attack multiple intakes can occur.

**Table 1:** Study results

Migraine frequency	
Average number of monthly migraine attacks	9.47
Primary outcome measure	
Mean number of acute anti-migraine drugs avoided per month per subject (lower bound)	
Total population (*n* = 413)	2.93
Attack users (*n* = 366)	3.31
Secondary outcome measures	
Percentage of subjects using the device to treat attacks	88.6%
Percentage of attacks treated with the device	71.8%
Percentage of Cefaly^®^-treated attacks for which acute anti-migraine drug intake is reduced	42.6%
Other results	
Proportion of drug classes with reduced intake	
Triptans	54.9%
Analgesics/NSAIDs	64.9%
Others	10.7%
Percentage of subjects unable to reduce acute medication intake in any of their attacks	18.3%
Reasons for not using Cefaly^®^ to treat migraine attacks	
I cannot bear the feeling during an attack	14.9%
It does not provide sufficient relief	48.9%
I never tried	10.6%
I do not have the device with me during an attack	12.8%
Others	12.8%

Half of subjects who did not use the Cefaly^®^ during an attack claimed this was due to a lack of efficacy; 23.4% did not use it for practical reasons and only 14.9% because of unbearable sensations due to the electrical stimulation.

All respondents were regular Cefaly^®^ users and the survey was thus biased towards subjects who were globally satisfied with the device. However, subjects were not informed beforehand that the focus of the survey was on attack treatment.

Clinical practice indicates that many migraine patients who purchased the Cefaly^®^ also use it during attacks, but that 88.6% of them would do so was not expected. This may be due to the user manual that recommends program 1 for attack treatment, besides program 2 for prevention. According to our survey, program 1 allows reducing specific and non-specific acute migraine drug consumption in 42.6% of attacks in more than 80% of subjects. Admittedly, this is not a direct measure of the effect of Cefaly^®^ on migraine attacks, but the high proportion of ictal users and attacks treated per subject (71.8%) suggests that the device is consistently beneficial. Moreover, the reduction of acute medication intake of 3.31 per subject per month has pharmaco-economic importance and reduces the chronifying risk of medication overuse in patients with frequent migraine.

Taken together, this survey suggests that e-TNS with Cefaly^®^ (program 1) may mitigate migraine attacks in subjects using regularly the device for prevention, as it is able to reduce intake of acute migraine drugs. It also indicates that Cefaly^®^ is well tolerated during an attack by the majority of subjects. These results need to be confirmed in a randomized, controlled trial.
